# Spirituality as an Essential Element of Person-Centered Care

**DOI:** 10.1093/geroni/igab046.3200

**Published:** 2021-12-17

**Authors:** Rachel Nathan, Deborah Zuercher, Steven Eveland, Anjana Chacko, Raya Kheirbek

**Affiliations:** 1 University of Maryland School of Medicine, University of Maryland, Maryland, United States; 2 University of Maryland Medical Center, University of Maryland, Maryland, United States; 3 University of Maryland, Baltimore, Maryland, United States; 4 University of Maryland School of Medicine, University of Maryland School of Medicine, Maryland, United States

## Abstract

Data demonstrate that the majority of patients with serious or chronic illness would like their clinicians to address their spirituality but that the majority of clinicians do not provide such care. Reasons cited include lack of training. Palliative Medicine, built on the biopsychosocial-spiritual model of care, has long recognized the critical role of spirituality in the care of patients with complex, serious, and chronic illness. There is mounting evidence that spiritual care is a fundamental component of all high-quality compassionate health care, and it is most effective when it is recognized and reflected in the attitudes and actions of both patients and health care providers. We conducted focus groups as a first step in the process to arrive at a consensus definition of “spiritual care.” A second step involved collecting and comparing frameworks and models that recognize that providers cannot be made compassionate simply through the imposition of rules; methods were needed to achieve behavior change. The study group developed and piloted curriculum to train health care providers. The created curricula covered the definitions of a spiritual care, self-awareness, cultural sensitivity, assessment, and skills. As part of ongoing curriculum development processes, training included evaluation tools to accompany skill development . Our work demonstrated the need for compassionate presence during encounters, for applying the spirituality in professional life; and for identifying ethical issues in inter-professional spiritual care. We concluded that it is feasible to train clinicians to address spirituality and provide holistic and patient-centered care in an effort to minimize suffering.

